# Supplementary material for: Recent Insights into Sample Pretreatment Methods for Mycotoxins in Different Food Matrices: A Critical Review on Novel Materials
Source: Toxins (Basel). 2023 Mar 10;15(3):215. doi: 10.3390/toxins15030215 (PMC10053610; doi:10.3390/toxins15030215)
Supplement: Supplementary file 1 [file toxins-15-00215-s001.zip › toxins-2261295-supplementary.pdf]

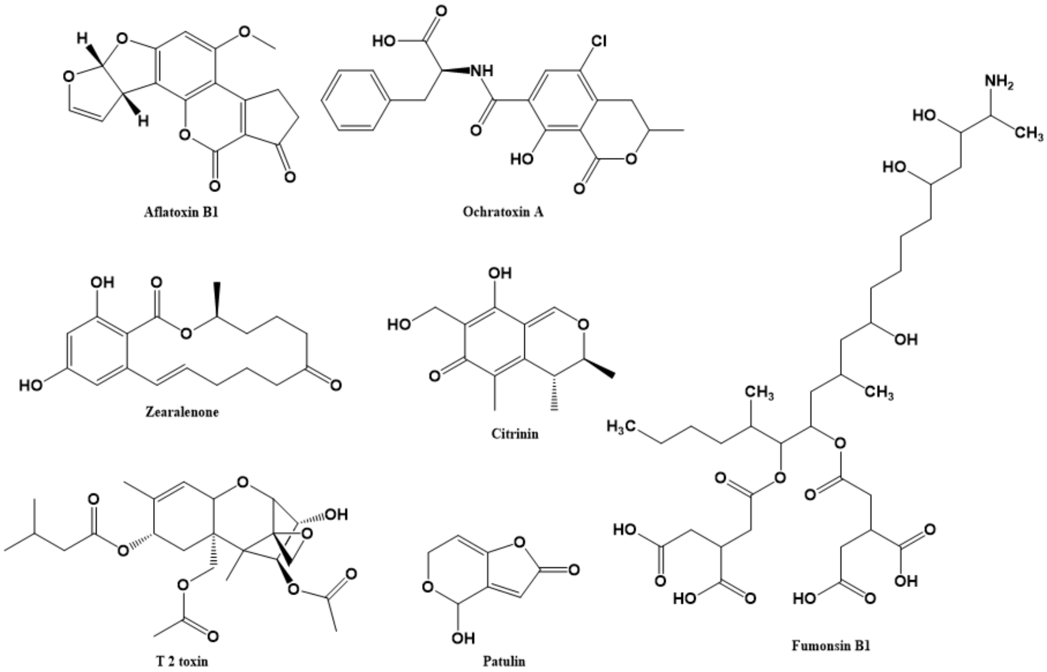

Figure S1. Chemical structure of major mycotoxins.

Table S1. The main classification of mycotoxins, representative toxins of each type and their toxicological effects.

Main Classification	Representative Toxins	Effect on Mammalian	Ref.
AFs	AFB1, AFB2, AFG1, AFG2, and AFM1	Carcinogenic, acute hepatitis, impaired immune system	[5]
CIT	CIT	Nephrotoxic	[2]
FBs	FB1 and FB2	Carcinogenic, hepatotoxic, causative agent in leukoencephalomalacia in horses	[6]
TCTs	T-2 and HT-2	Immuno-depressants, gastrointestinal haemorrhaging	[7]
OTs	OTA	Carcinogenic, nephrotoxic, hepatotoxic, teratogenic	[8]
PAT	PAT	Lung and brain haemorrhaging, carcinogenic, immunotoxic, genetoxic	[9]
ZEA	ZEA and its five metabolites α-zearalenol (α-ZEL), β-ZEL, α-zearalanol (α-ZAL), β-ZAL, and zearalanone (ZAN)	Estrogenic activity, potential carcinogenic and teratogenic	[10]

Table S2. Sample pretreatment methods used for mycotoxins since 2017.

Matrix	Targets	Pretreatment Methods	Ref.
rice	AFB1, B2, G1, G2	SLE	[16]
rice	AFB1, B2, G1, G2	SLE-DES	[17]
rice and fragrant rice	AFB1, B2, G1, G2	SLE-SPE	[18]
rice and noodle products	AFB1, B2, G1, G2	SPE	[19]
rice and wheat	AFB1, B2, G1, G2	SPE	[20]
rice and sorghum	AFB1, B2, G1, G2	MSPE	[21]
rice and maize	AFB1, B2, G1, G2	MSPE	[22]

rice, edible oil and milk	AFB1, B2, G1, G2	MSPE	[23]
corn, rice and millet	AFB1, B2, G1, G2	MSPE	[24]
rice grain	AFB1, Sterigmatocystin, ZEA	IT-SPME	[25]
rice bran	13 mycotoxins	DLLME	[26]
rice	AFB1, B2, G1, G2	AA-DLLME	[27]
rice	AFB1, B2, G1, G2	VALDS-ME	[28]
rice	AFs	MSPE	[29]
cereal products	HT-2 and T-2	SPE	[30]
cereals	AFB1, B2, G1, G2	SPE	[31]
cereal matrices	6 TCTs	SWE-SPE	[32]
cereals	AFB1, B2, G1, G2	MSPE	[33]
cereals	AOH, AME, ALT, TEN, and TeA	QuEChERS-DLLME	[34]
cereals	DON and ZEA	QuEChERS	[35]
corn and peanut	AFB1	SPE	[36]
corn meal	AFB1, B2, G1, G2, FB1, and ZEA	SLE	[37]
corn and corn products	FB_1 and FB_2	SPE	[38]
corn	ZEA and its derivatives	SPE	[39]
corn powder	FB_1	syringe SPE	[40]
maize	AFs, ochratoxins and enniatins	MSPE	[41]
maize	ZEA and its derivatives	MSPE	[42]
maize	FB_1 and FB_2	MSPD	[43]
maize and oats	DON, T-2	SPE	[44]
maize, wheat, watermelon and melon	AFB1, B2, G1, G2, OTA, OTB, T-2, HT-2 and DAS	MSPE	[45]
wheat and peanut	AFB1, B2, G1, G2	D-μ-SPE	[46]
wheat	AFB1, B2, G1, G2	DLLME	[47]
wheat	AFs	IL-DLLME	[48]
wheat and maize	AFB1, B2, G1, G2, OTA, STE, FB1, FB2, T-2, DON, and ZEA	QuEChERS	[49]
cornmeal	AFB1, B2, G1, G2	MSPD	[50]
cornmeal	ZEN	MSPE	[51]
infant consumption cereal-based porridge	14 mycotoxins	UAE	[52]
cereal-derived products	AFs, T-2 and HT-2 toxins, and fumonisins	QuEChERS	[53]
soybean	AFB1, B2, G1, G2	in-syringe SPE-DLLME	[54]
peanut	AFB1	SPME	[55]
peanut, almond and pistachio	16 mycotoxins	QuEChERS	[56]
fresh peanut	AFB1	UAE	[57]
peach seed, milk powder, corn flour and beer	FB1, AFB1, OTB, T-2 toxin, OTA and ZEA	MA-d-μ-SPE	[58]
biscuit	9 mycotoxins	QuEChERS	[59]
foodstuffs	AOH and AME	SPE	[60]
foodstuffs	AFB1, B2, G1, G2, AFM1, and AFM2	MSPE	[61]
peanut oils	AFB1	LLE-SPE	[62]
edible oils	AFB1, B2, G1, G2	LLE-SPE	[63]
edible vegetable oils	AFB1, B2, G1, G2	MSPE	[64]
vegetable oil	FB1, ZON and OTA	MSPE	[65]
vegetable oils	AFB1, B2, G1, G2	MSPE	[66]
edible oil, soy sauce and bean sauce	12 mycotoxins	SPE	[67]
milk powders	AFB1, B2, G1, G2 and M1	SPE	[68]
milk and dairy products	AFM1	SPE	[69]
milk	AFM1	SPE	[70]

milk	9 mycotoxins	SPE	[71]
milk and yogurt	6 mycotoxins	MSPE	[72]
milk	AFM1	SALLE-on-line SPE	[73]
soy milk	AFB1, B2, G1, G2	D-μ-SPE	[74]
milk	AFM1	DES-DLLME	[75]
egg and milk	39 mycotoxins and metabolites	QuEChERS	[76]
functional vegetable milks	FB1 and B2, HT-2 and T-2, ZEA, DON and fusarenon-X	SALLE	[77]
yogurt	AFs	DLLME	[78]
cheese	32 mycotoxins	QuEChERS	[79]
nuts	OTA, AFB1, B2, G1, G2	dilute-and-shoot	[80]
nut	AFB1, B2, G1, G2	SPE	[81]
nuts	AFB1, B2, G1, G2	MSPE	[82]
Pistachio nuts	AFB1, B2, G1, G2, OTA, ZEA, T2, and HT2	QuEChERS	[83]
chestnut	14 mycotoxins	d-SPE-QuEChERS	[84]
fruits and vegetables	7 mycotoxins	SPE	[85]
apple products and dried fruits	patulin	VA-DSPME	[86]
apple juice	patulin	SD-LLLME	[87]
fruit berry by-products	AFs, OTA and Alternaria toxins	QuEChERS	[88]
grapes, processed grape products and animal-derived products	OTA	QuEChERS	[89]
strawberries	patulin	QuEChERS	[90]
red-pigmented fruits	patulin	QuEChERS	[91]
button mushroom	13 mycotoxins	Dilute-and-shoot	[92]
tomato-based and fruit-based products	AOH, AME, TeA, and TEN	QuEChERS	[93]
tomatoes and derived tomato products	AME, AOH, TEN, TeA, ALT, ENNs A, A1, B, and B1, and BEA	QuEChERS	[11]
fruits, vegetables and their derivatives marketed	AOH	QuEChERS	[94]
chili pepper and processed groundnut	AFB1, B2, G1, G2	SPME	[95]
feed	FB_1 and FB_2	SLE-SPE	[96]
animal feed and food	11 mycotoxins	SLE-SPE	[97]
fish feed	AFB1, B2, G1, G2	μ-SPE	[98]
feed ingredients and compound feeds	11 Mycotoxins	QuEChERS	[99]
mixed feed rations	25 mycotoxins	(d)SPE-QuEChERS	[100]
feed	15 mycotoxins	QuEChERS	[101]
animal feeds	ENNs and BEA	QuEChERS	[102]
feed and foodstuffs	CIT and OTA	QuEChERS	[103]
beer, red wine, corn, and Turkish coffee	OTA	SPE	[104]
beer	ZEA	on-line SPE	[105]
wine	OTA	on-line SPE	[106]
beer	23 mycotoxins	QuEChERS	[107]
dark tea	AFB1, B2, G1, G2	MFC-IAC	[108]
dark tea	AFB1, B2, G1, G2	QuEChERS	[109]
Pu-erh tea	AFB1	QuEChERS	[110]
coffee beans	17 mycotoxins	QuEChERS	[111]
cocoa beans	ochratoxin A and AFB1, B2, G1, G2	QuEChERS	[112]
non-dairy beverages	AFB1, B2	μ-SPE	[113]

plant-based beverages	AFB1, B2, G1, G2, OTA and DON, ZEA, T-2, HT-2, FB1 and FB2	QuEChERS	[114]
edible and medicinal herbs	6 AFs and 6 ZEAs	SPE	[115]
Salviae miltiorrhiza Radix et Rhizoma (Danshen)	ZEA, T-2, HT-2, NEO, DAS	MSPE	[116]
Indian medicinal herbs	AFB1, B2, G1, G2	QuEChERS	[117]
functional and medicinal herbs	AFB1, B2, G1, G2, OTA, ZEA, DON, FB1, B2, B3 and T-2	SPE (IAC)-QuEChERS	[118]
rat faeces	DON, 3AcDON, 15AcDON, DON3G, and DOM-1	QuEChERS-d-SPE	[119]
human urine	ZEA, α-ZEL, β-ZEL, α-ZAL, β-ZAL, ZAN	SPE	[120]
pig hair	FB_1	SLE-SPE	[121]
chicken liver	DON, 3-ADON, 15-ADON, NIV, FUS-X, NEO, HT-2 and T-2	QuEChERS	[122]
endometrial cancer tissues	ZEA	QuEChERS	[123]
